# Verticillin A Inhibits Leiomyosarcoma and Malignant Peripheral Nerve Sheath Tumor Growth via Induction of Apoptosis

**DOI:** 10.4172/2161-1459.1000221

**Published:** 2016-10-24

**Authors:** A Zewdu, G Lopez, D Braggio, C Kenny, D Constantino, HK Bid, K Batte, OH Iwenofu, NH Oberlies, CJ Pearce, AM Strohecker, D Lev, RE Pollock

**Affiliations:** 1 Department of Surgical Oncology, Ohio State University Wexner Medical Center, Columbus, Ohio, USA; 2 The James Cancer Center, Ohio State University Wexner Medical Center, Columbus, Ohio, USA; 3 Resonant Therapeutics, Inc., Ann Arbor, Michigan, USA; 4 Department of Pathology, Comprehensive Cancer Center, Ohio State University, Columbus, Ohio, USA; 5 Department of Chemistry and Biochemistry, University of North Carolina at Greensboro, Greensboro, North Carolina, USA; 6 Mycosynthetix, Inc., Hillsborough, North Carolina, USA; 7 Department of Cancer Biology and Genetics, Ohio State University, Columbus, Ohio, USA; 8 Surgery B, Sheba Medical Center, Tel Aviv, Israel

**Keywords:** Verticillin A, Epipolythiodioxopiperazine alkaloid, Malignant peripheral nerve sheath tumor, MPNST, Leiomyosarcoma, LMS, Apoptosis, Soft tissue sarcoma, STS

## Abstract

**Objective:**

The heterogeneity of soft tissue sarcoma (STS) represents a major challenge for the development of effective therapeutics. Comprised of over 50 different histology subtypes of various etiologies, STS subsets are further characterized as either karyotypically simple or complex. Due to the number of genetic anomalies associated with genetically complex STS, development of therapies demonstrating potency against this STS cluster is especially challenging and yet greatly needed. Verticillin A is a small molecule natural product with demonstrated anticancer activity; however, the efficacy of this agent has never been evaluated in STS. Therefore, the goal of this study was to explore verticillin A as a potential STS therapeutic.

**Methods:**

We performed survival (MTS) and clonogenic analyses to measure the impact of this agent on the viability and colony formation capability of karyotypically complex STS cell lines: malignant peripheral nerve sheath tumor (MPNST) and leiomyosarcoma (LMS). The in vitro effects of verticillin A on apoptosis were investigated through annexin V/PI flow cytometry analysis and by measuring fluorescently-labeled cleaved caspase 3/7 activity. The impact on cell cycle progression was assessed via cytometric measurement of propidium iodide intercalation. *In vivo* studies were performed using MPNST xenograft models. Tumors were processed and analyzed using immunohistochemistry (IHC) for verticillin A effects on growth (Ki67) and apoptosis (cleaved caspase 3).

**Results:**

Treatment with verticillin A resulted in decreased STS growth and an increase in apoptotic levels after 24 h. 100 nM verticillin A induced significant cellular growth abrogation after 24 h (96.7, 88.7, 72.7, 57, and 39.7% reduction in LMS1, S462, ST88, SKLMS1, and MPNST724, respectively). We observed no arrest in cell cycle, elevated annexin, and a nearly two-fold increase in cleaved caspase 3/7 activity in all MPNST and LMS cell lines. Control normal human Schwann (HSC) and aortic smooth muscle (HASMC) cells displayed higher tolerance to verticillin A treatment compared to sarcoma cell lines, although toxicity was seen in HSC at the highest treatment dose. *In vivo* studies mirrored the in vitro results: by day 11, tumor size was significantly reduced in MPNST724 xenograft models with treatment of 0.25 and 0.5 mg/kg verticillin A. Additionally, IHC assessment of tumors demonstrated increased cleaved caspase 3 and decreased proliferation (Ki67) following treatment with verticillin A.

**Conclusion:**

Advancement in the treatment of karyotypically complex STS is confounded by the high level of genetic abnormalities found in these diseases. Consequently, the identification and investigation of novel therapies is greatly needed. Our data suggest that verticillin A selectively inhibits MPNST and LMS growth via induction of apoptosis while exhibiting minimal to moderate effects on normal cells, pointing to verticillin A as a potential treatment for MPNST and LMS, after additional preclinical validation.

## Introduction

STS is a rare cancer of mesenchymal origin accounting for less than 1% of adult solid malignancies. This tumor cluster encompases over 50 subtypes, ranging from the highly metastatic (e.g., Ewing sarcoma) to those unable to metastasize from the primary site of development (e.g., desmoid tumor) [1]. These STS subsets are further characterized as genetically simple or complex cancers based on the presence or absence of fusion proteins as well as chromosome abnormalities [2,3]. Overall, STS patients experience high risks of local recurrence, distant metastasis, and unacceptably low disease-free survival [4–6], especially in the case of high grade STS. This reality points to the lack of effective systemic treatment options for patients. Conventional STS treatment includes chemotherapy and radiation therapy, as well as total surgical excision, all of which can have serious quality of life consequences [4]. Drug combinations, such as doxorubicin and ifosfamide, are commonly used in the treatment of these malignancies, albeit with modest efficacy:toxicity ratios [4–8].

Multiple chromosomal abnormalities including regional mutation, deletion, and amplification events render the effective treatment of complex karyotype STS extremely difficult [2,9]; the utilization of multiple aberrantly regulated pathways by these genetically complex malignancies not only complicates the development of targeted therapies but also increases the likelihood of therapeutic resistance. Identification of systemic therapy exhibiting selective potency with minimal patient toxicity is therefore critical.

Verticillin A is a small molecule member of a large and diverse family of epipolythiodioxopiperazine (ETP) alkaloids [10]. Identified as a fungal toxin released in response to pathogen infection, the anticancer capacity of verticillin A has been observed in metastatic colon carcinoma [11,12]. Although verticillin A displayed high efficacy as a single agent in this disease, combining verticillin A with TRAIL in a treatment approach resulted in both the potentiation of TRAIL as well as the reduction in the concentration of verticillin A required for demonstrable effect, hence our interest in evaluating the utility of this compound in STS.

## MATERIALS AND METHODS

### Reagents and drugs

Verticillin A was purified from culture MSX59553 from the Mycosynthetix fungal library, as described in detail previously [10]; the sample was re-isolated from MSX59553 and was >95% pure as measured by UPLC. Aliquots of verticillin A were reconstituted in dimethyl sulfoxide (DMSO; Fisher Bioreagents, Pittsburg, PA, USA) for in vitro studies or cremophor EL (Sigma-Aldrich, St. Louis, MO, USA) for *in vivo* investigations, and stored at −20° C. Antibodies (cleaved caspase 3, ^#^9661S and Ki67, ^#^VP-K451) used for immunohistochemistry (IHC) assessment were purchased from Cell Signaling (Danvers, MA, USA) and Vector Laboratories (Burlingame, CA, USA), respectively, and used at a dilution of 1:1000. Propidium iodide (PI; ^#^P4864-10ML) was obtained from Sigma-Aldrich (St. Louis, MO, USA).

### Cell culture and cell lines

Human NF1-associated MPNST cell lines, ST88 and S462, and sporadic MPNST cell line, MPNST724, have been previously described [13]. MPNST724 and ST88 were attained from Dr. Jonathan Fletcher (Brigham and Women’s Hospital, Boston, MA, USA), and S462 was acquired from Dr. Lan Kluwe (University Hospital Eppendorf, Hamburg, Germany). LMS cell lines SKLMS1 and LMS1 were acquired from ATCC (Manassas, VA, USA) and Dr. Dominique Broccoli (Mercer University, Savannah, GA, USA), respectively. Cells were cultured in complete DMEM (DMEM with sodium pyruvate, L-glutamine high glucose, 10% FBS and 100 µg/mL Normacin) and maintained at 37° C at 5% CO_2_ for the duration of the experiments. Human Schwann (HSC) and aortic smooth muscle (HASMC) cells (ScienCell, Carlsbad, CA, USA) were cultured in the appropriate media as described by supplier protocol and used as controls for in vitro assays. Primocin, used to supplement normal cell media, and Normacin, added to sarcoma cell media, were purchased from Invitrogen (Carlsbad, CA, USA). DMEM (^#^11995-065) was purchased from Thermo-Fisher (Grand Island, NY).

### Cell viability analysis

MTS analysis was performed using CellTiter96 Aqueous Non-Radioactive Cell Proliferation Assay kit (Promega, Madison, WI, USA) as specified by the manufacturer. Cells were seeded at a density of 5,000 cells per well, and allowed to adhere overnight. Cellular viability was assessed 24 h after treatment with DMSO (control), or 10, 50, or 100 nM verticillin A, and absorbance was measured at 490 nm wavelength.

### Clonogenic analysis

Cells were seeded at 800 cells per well in a 6-well plate (9.5 cm^2^) and allowed to attach overnight prior to treatment with DMSO or varying concentrations of verticillin A. Cells were treated for 24 h, then changed to recovery media and allowed to continue to grow for 10 days. Colonies were stained using 0.5% crystal violet solution in methanol for 30 min. Staining solution was removed, wells were washed with deionized H_2_O, and stained colonies were imaged and counted.

### Apoptosis analysis

Apoptosis was measured by quantifying cleaved-caspase 3 and cleaved-caspase 7 activity using Cell Event Caspase 3/7 Green Detection Reagent (Life Technologies, Carlsbad, CA, USA) in the Incucyte Zoom system (Essen BioScience, Ann Arbor, MI, USA). Cells were plated at a density of 4,200 cells per well and allowed to adhere. The cells were then treated with DMSO or 10, 50, or 100 nM verticillin A for 24 h, and caspase activity was monitored using fluorescent Cell Event Caspase 3/7 Reagent (5 µM final concentration). Endpoint analysis was performed in each well using a final concentration of 10 µM Vybrant DyeCycle Green Stain (Life Technologies, Carlsbad, CA, USA) in complete DMEM. Apoptosis was confirmed using the Apoptosis Detection Kit (BD Pharmagen, San Diego, CA, USA) in complete DMEM. Per supplier instructions, 1 × 106 cells/mL were stained with 5 µL Annexin V-FITC and 5 µL propidium iodide following 24 h treatment with DMSO or 10, 50, or 100 nM verticillin A and analyzed via FACS analysis (LSR II, BD Pharmagen, San Diego, CA, USA).

### Cell cycle analysis

Cells were synchronized by 16 h starvation prior to treatment with DMSO, 10, 50, or 100 nM of verticillin A for 24 h. Attached and floating cells were collected following treatment and centrifuged for 5 min at 2500 RPM. Supernatant was removed and cells were washed twice with cold PBS. Cells were centrifuged (2500 RPM/5 min) and resuspended in 70% ethanol and fixed overnight at −20° C. Cells were then centrifuged at (2500 RPM/5 min), and ethanol was removed. Subsequently, cells were resuspended in 400 µL PI stain (0.05 mg/mL/ 1 mg/mL sodium citrate/ 50 µL Triton-X-100/ 50 µL RNase A, brought to 50 mL with PBS) and analyzed via LSR II FACS analysis.

### *In vivo* animal models

Six week old female SCID mice (Taconic Biosciences, Hudson, NY, USA; model ^#^CB17SC) were injected subcutaneously with 1 × 106 MPNST724 cells into the flank. Once tumors reached 0.5 cm, mice were allocated into three arms (10 mice per arm) and treatment via intraperitoneal injections was initiated: Vehicle (5% ethanol/15% cremophor EL/80% PBS), verticillin A (0.25 mg/kg every other day), or verticillin A (0.5 mg/kg every other day). Mice were weighed and tumors were measured twice weekly. Mice were euthanized once tumors in the control group grew to ~1.5 cm. Final tumor volumes and weights were measured, and tumors were processed for IHC analysis.

### Immunohistochemistry and H&E analyses

Ki67 (Vector Laboratories) and cleaved caspase 3 (Cell Signaling) antibodies were used to identify the effect of verticillin A on proliferation and apoptosis, respectively. IHC staining was conducted at Nationwide Children’s Hospital (Columbus, OH) and analysis was performed at the Polaris Innovation Center (The Ohio State University, Columbus, OH, USA). IHC samples and analyses were verified by pathologist, O. Hans Iwenofu, M.D. (The Ohio State University, Department of Pathology, Columbus, OH, USA).

### Statistics

*In vitro* analyses were performed in triplicate. Mean ± SEM (standard error mean) calculations and statistical analyses were performed for all cell-based assays, and EC50 values were computed using GraphPad Prism version 6.00 (for Windows, GraphPad Software, La Jolla California USA, www.graphpad.com). All Student t tests performed were unpaired and two-sided. The average tumor volume (mm^3^) and weight (kg) for each study arm was measured and recorded. Mean ± SEM was calculated for each treatment group, and end-point analyses determined using unpaired two-sided t test was utilized to determine variances. *p ≤ 0.05; **p ≤ 0.01; ***p ≤ 0.001.

## Results

### Verticillin A affects STS cellular viability and colony formation capability

**Figure 1:**
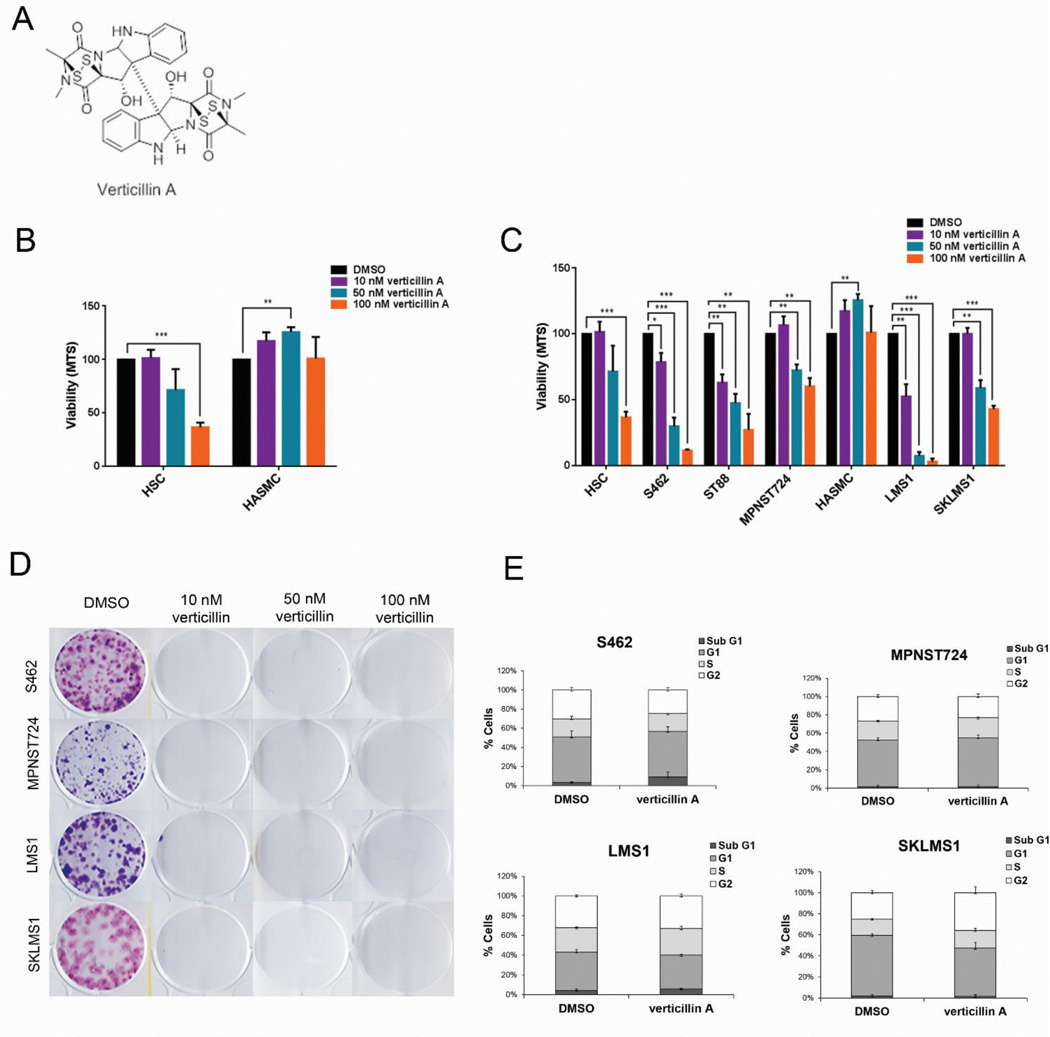
STS treated with verticllin A demonstrate decreased cell viability in vitro. (A) Molecular structure of verticillin A. (B) MTS analysis of normal cells (HASMC and HSC) treated with verticillin A at indicated doses for 24 h. (C) MTS assessment of leiomysarcoma (SKLMS1 and LMS1) and MPNST (MPNST724 and S462) cell lines treated increasing concentrations of verticillin A for 24 h. (D) Analysis of colony formation capability of S462, MPNST724, LMS1, and SKLMS1 upon treatment. (E) Cell cycle progression. *p<0.05, **p<0.01, ***p<0.001.

To assess the effect of verticillin A on STS, genetically complex MPNST and LMS cell lines were treated with increasing doses of verticillin A (Figure 1A). Primary cultured human Schwann (HSC) and smooth muscle (HASMC) cells were used to evaluate the effects of this compound on normal control cells. Verticillin A showed no effect on HASMC at 100 nM and a displayed potency in HSC (EC50=64.94 nM) (Table 1) (Figure 1B). Following 24 h of treatment, verticillin A demonstrated a marked inhibitory effect on S462, ST88, and LMS1 at 10 nM while MPNST724 and SKLMS1 exhibited tolerance at this dose (Figure 1C). All STS cell lines displayed a significant decrease in viability with 100 nM verticillin A. The effects elicited in these complex karyotype STS cell lines, as compared to normal cells, suggest the possible selective inhibitory capacity of these agents.

Verticillin A also inhibited colony formation capacity in STS cells; with all doses of verticillin A, all MPNST and LMS cell lines were unable to form colonies (Figure 1D). We next investigated whether the response of these STS cells was due to a compound-mediated cell cycle arrest. No substantial arrest in cell cycle progression was demonstrable in MPNST or LMS (Figure 1E), indicating that anticancer effect of verticillin A is independent of cycle arrest (Figure 1).

### Verticillin A induces apoptosis in MPNST and LMS

**Figure 2:**
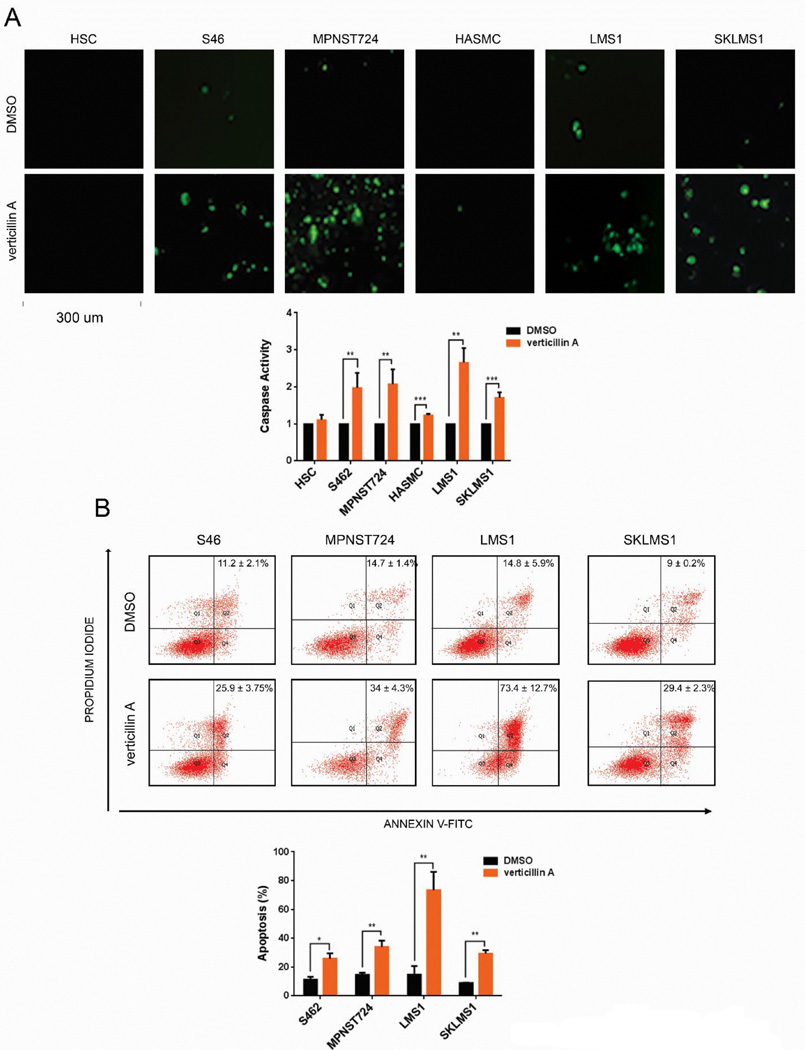
Verticillin A induces apoptosis in STS. (A) Cleaved caspase 3/7 activity as measured by time lapse microscopy. Representative images are shown. (B) Annexin V/PI FACS analysis of apoptotic levels.

Published works indicate that verticillin A impedes tumor growth via stimulation of apoptotic machinery [11]. Therefore, induction of apoptosis in STS was initially explored as a potential mechanism of action. Cleaved caspase 3 and 7 activities, markers of apoptosis, were measured using fluorescent Cell Event Caspase 3/7 Reagent. When activated, these caspases recognize the caspase-3/7 recognition motif (DEVD) on the Caspase 3/7 Reagent and cleave the substrate, thereby releasing the fluorescent DNA intercalating dye. The dye then binds DNA and produces a measurable signal. All STS cell lines displayed increased caspase activity whereas the effects on HSC and HASMC (1.1 and 1.2 fold change, respectively) were modest (Figures 2A). Annexin V/PI staining further supported this finding. All STS cell lines, but not normal control cells, exhibited significantly increased apoptosis upon verticillin A treatment (LMS1, 73.4 ± 12.7%; MPNST724, 34 ± 4.3%; SKLMS1, 29.4 ± 2.3%; S462, 25.9 ± 3.75%) (Figure 2B). Taken together, these data suggest that verticillin A inhibits STS growth via induction of apoptosis (Figure 2).

### Verticillin A reduces tumor growth *in vivo*

**Figure 3:**
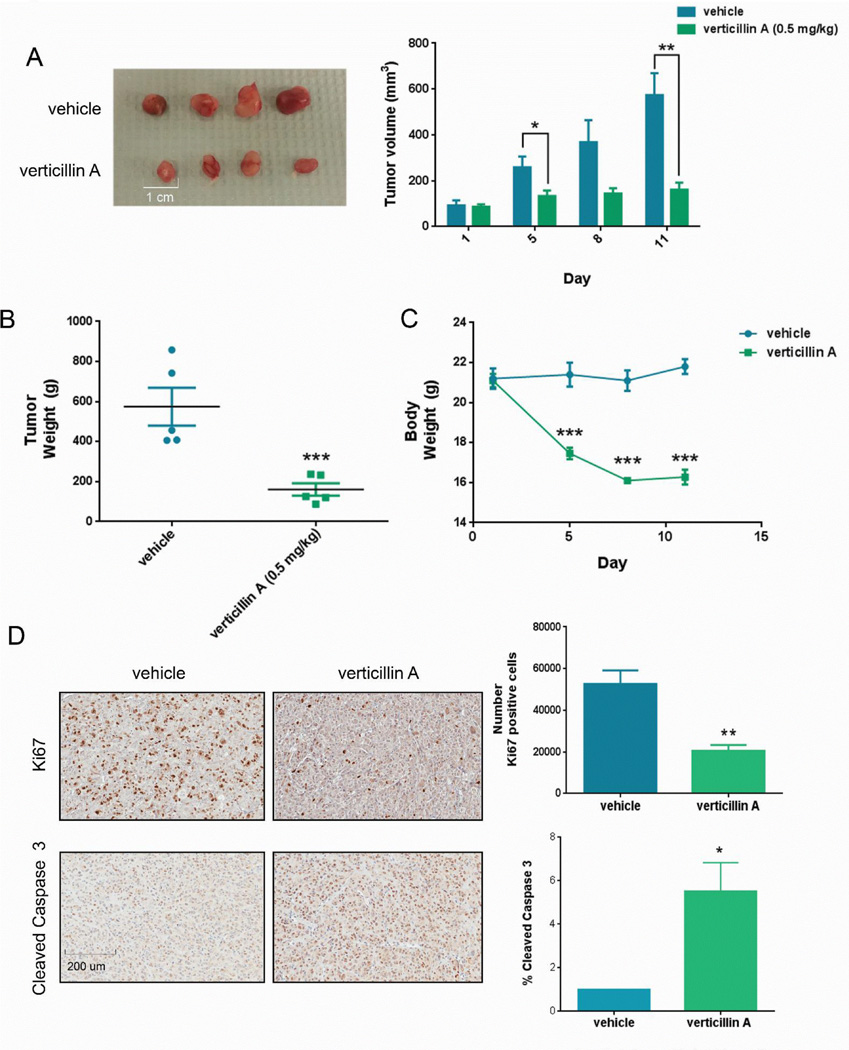
Verticillin A inhibits MPNST724 growth *in vivo*. (A and B.) Gross histology of tumors removed from mice treated with either vehicle or 0.5 mg/kg verticillin A at day 11. (B) Tumor burden by day 11 (p<0.0001). (C) Mouse body weight. (D) IHC analysis of proliferation (Ki67, p=0.015) and apoptosis (cleaved caspase 3, p=0.042) upon treatment with 0.5 mg/kg verticillin A treatment.

Next, the effects of verticillin A on STS tumor growth *in vivo* was investigated via use of MPNST724 xenografts. MPNST724 cells demonstrated higher tolerance to verticillin A (EC50=124.6 nM). It was therefore predicted that any efficacy seen in the MPNST724 xenograft models would be amplified in the other STS lines, as based on our EC50 data. MPNST724 xenografts demonstrated significant tumor growth inhibition in the mice allocated to either verticillin A treatment arms with the greatest effects observed with 0.5 mg/kg drug (tumor volume=160.92 mm^3^, p ≤ 4.05E-06; tumor weight=0.2g, p ≤ 6.77E-05) (Figure 3A–C); treatment with 0.25 mg/kg also significantly reduced tumor volume (355.89 mm^3^, p ≤ 0.000147) by day 11 (Supplemental Figure 1).

Toxicity was observed in mice treated at the higher 0.5 mg/kg dose; mice demonstrated reduced grooming tendencies and mild weight loss (Figure 3D). Additionally, 2 mice were lost due to verticillin A-induced mortality. Further toxicology studies are therefore needed to understand the scope of these verticillin A-induced side effects.

Verticillin A also induced a nearly 40% decrease in the Ki67 proliferation marker expression in MPNST724 xenografts (Figure 3E). To identify compound-induced apoptosis *in vivo*, we probed for the cleaved caspase 3 apoptosis marker using IHC analysis. Verticillin A treatment led to a 5.5-fold increase in cleaved caspase 3 expression in MPNST724 tumors (Figure 3D). Taken together, the observed in vitro selectivity and *in vivo* inhibitory effect of verticillin A supports further evaluation of its potential as a useful anti-STS clinical agent (Figure 3).

## Discussion

Verticillin A is a small molecule with demonstrated anticancer activity via chromatin remodelling [10–12], although little is known on the effects of verticillin A on normal cells. Our data demonstrate the cytotoxic selectively of this agent toward STS cell lines compared to normal cells. While verticillin A impairs growth effects on these normal cells, their tolerance to verticillin A is greater than that of their STS counterparts in comparing HASMC to LMS. Likewise, Liu et al. demonstrated marked anticancer effects of verticillin A in colon carcinoma cell lines and higher tolerance in normal human colon epithelial cell (CCD-841) and donor T cells [11]. This selective responsiveness of cancer cells to verticillin A suggests the potential value of using these drugs in MPNST and LMS treatment.

Our data did not show cell cycle arrest in response to verticillin A; instead, verticillin A treatment resulted in an anticancer effect independent of cell cycle arrest. In contrast, verticillin A was shown to induce G2 cell cycle arrest in SW620 colon cancer cells, though no changes in cell cycle progression were observed in verticillin A-treated HepG2 liver carcinoma cells [11]. Taken together, these observations suggest that verticillin A may have various impacts on cell cycle progression depending on cell type being investigated, implying possible discrepancies in utilized pathways, differential metabolic processes, and differences in genetic and proteomic expression.

Minor signs of toxicity and compound-mediated mortalities were observed in the mice treated with the lower and higher doses of verticillin A, respectively. Further investigation is therefore needed to determine whether toxicity issues resolve following compound withdrawal. Toxicity levels may potentially be mitigated through a combinational therapeutic approach. In the exploration of combination TRAIL/verticillin A therapy on high grade colon carcinoma, Liu et al. were able to reduce the verticillin A dosage from 30–122 nM to 10 nM11, thereby successfully sensitizing tumor cells to TRAIL treatment. Additionally, studies performed by our laboratory have demonstrated an effect of broad-spectrum histone deacetylase inhibitors (HDACis) on genetically complex STS [14,15], and improved efficacy:toxicity ratio through HDAC isoform-specific targeting [16]. Given the observed efficacy demonstrated by HDACis and verticillin A as single agents, investigation of combined HDACis/ verticillin A therapeutic approaches may prove useful in the treatment of patients with high grade STS.

The study of the anti-tumor effects of natural compounds is a decades-long effort to identify more efficacious treatment strategies; between 1981 and 2010, 48.6% of all new drugs introduced during this time were marine-, microorganism-, or plant-derived, roughly 65% of which are anticancer compounds [17,18]. These discoveries thereby support the continued investigation of new and understudied natural compounds in hopes of identifying novel compounds with selective anticancer potency.

Many natural compounds, such as romidepsin [19], have been shown to display growth inhibitory capacities in STS [20,21]. Given the success seen in the in vitro investigation of other bioactive compounds, investigation of the natural product verticillin A in STS was warranted. Our exploration of verticillin A suggests the possible value of this agent in patient care; however, the results are still preliminary and will require further evaluation for the potential treatment of patients with MPNST and LMS.

## Supplementary Material

Suppl file

## Figures and Tables

**Table 1:** EC50 values of normal and STS cell lines were determined via MTS assessment after 24 h treatment with verticillin A.

Cell Line	EC50 (Nm)
HASMC	250.7
HSC	64.94
MPNST724	124.6
S462	26.03
SKLMS1	74.56
LMS1	10.69
