# Supplementary material for: Verticillin A Inhibits Leiomyosarcoma and Malignant Peripheral Nerve Sheath Tumor Growth via Induction of Apoptosis
Source: Clin Exp Pharmacol. Author manuscript; Available in PMC 2017 Feb 7. (PMC5295762; doi:10.4172/2161-1459.1000221)
Supplement: Suppl file [file NIHMS838698-supplement-Suppl_file.pdf]

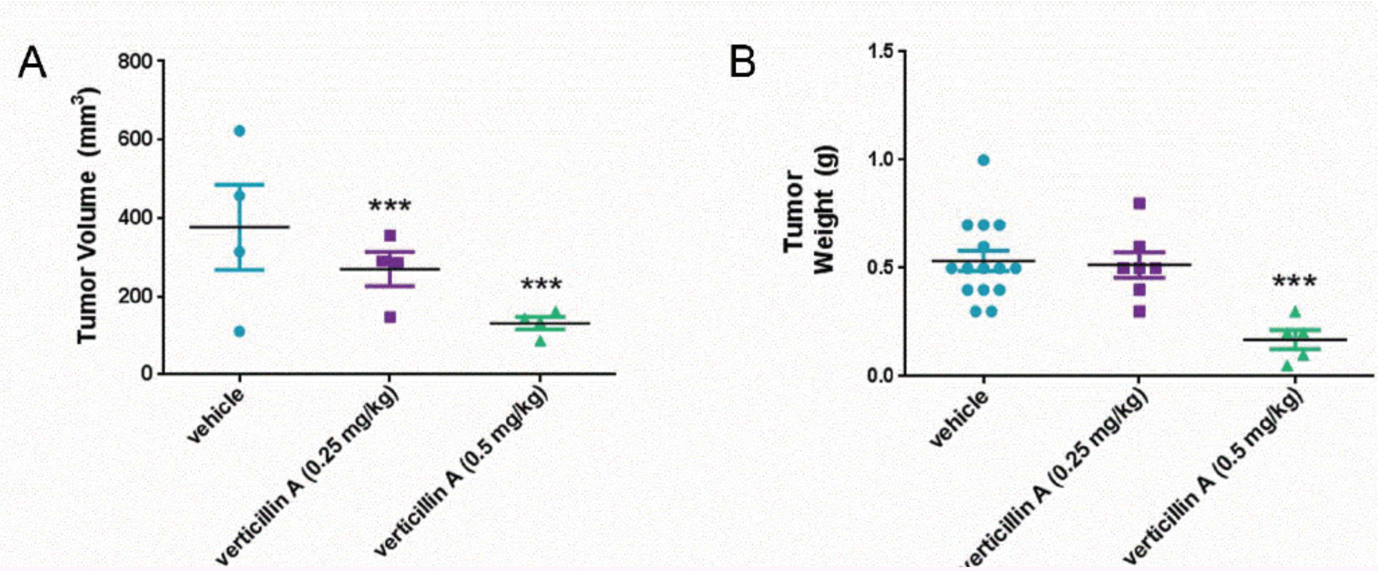

Supplementary Figure 1: Verticillin A inhibits tumor growth with 0.25 mg/kg treatment. A.) Endpoint measurement of tumor volumes at day 11 after treatment with vehicle, 0.25 or 0.5 mg/kg verticillin A.
